# A Placebo-Controlled Study on the Effects of the Glucagon-Like Peptide-1 Mimetic, Exenatide, on Insulin Secretion, Body Composition and Adipokines in Obese, Client-Owned Cats

**DOI:** 10.1371/journal.pone.0154727

**Published:** 2016-05-02

**Authors:** Kirsten M. Hoelmkjaer, Nicolai J. Wewer Albrechtsen, Jens J. Holst, Anna M. Cronin, Dorte H. Nielsen, Thomas Mandrup-Poulsen, Charlotte R. Bjornvad

**Affiliations:** 1 Department of Veterinary Clinical and Animal Sciences, Faculty of Health and Medical Sciences, University of Copenhagen, Frederiksberg, Denmark; 2 NNF Center for Basic Metabolic Research, Faculty of Health and Medical Sciences, University of Copenhagen, Copenhagen, Denmark; 3 Immuno-endocrinology laboratory, Department of Biomedical Sciences, Faculty of Health and Medical Sciences, University of Copenhagen, Copenhagen, Denmark; INIA, SPAIN

## Abstract

Glucagon-like Peptide-1 mimetics increase insulin secretion and reduces body weight in humans. In lean, healthy cats, short-term treatment has produced similar results, whereas the effect in obese cats or with extended duration of treatment is unknown. Here, prolonged (12 weeks) treatment with the Glucagon-like Peptide-1 mimetic, exenatide, was evaluated in 12 obese, but otherwise healthy, client-owned cats. Cats were randomized to exenatide (1.0 μg/kg) or placebo treatment twice daily for 12 weeks. The primary endpoint was changes in insulin concentration; the secondary endpoints were glucose homeostasis, body weight, body composition as measured by dual-energy x-ray absorptiometry and overall safety. An intravenous glucose tolerance test (1 g/kg body weight) was conducted at week 0 and week 12. Exenatide did not change the insulin concentration, plasma glucose concentration or glucose tolerance (P>0.05 for all). Exenatide tended to reduce body weight on continued normal feeding. Median relative weight loss after 12 weeks was 5.1% (range 1.7 to 8.4%) in the exenatide group versus 3.2% (range -5.3 to 5.7%) in the placebo group (P = 0.10). Body composition and adipokine levels were unaffected by exenatide (P>0.05). Twelve weeks of exenatide was well-tolerated, with only two cases of mild, self-limiting gastrointestinal signs and a single case of mild hypoglycemia. The long-term insulinotropic effect of exenatide appeared less pronounced in obese cats compared to previous short-term studies in lean cats. Further investigations are required to fully elucidate the effect on insulin secretion, glucose tolerance and body weight in obese cats.

## Introduction

Glucagon-like Peptide-1 (GLP-1) is a protein secreted from the L-cells in the distal small intestine in response to the presence and absorption of nutrients in the gut lumen and GLP-1 amplifies glucose-induced insulin secretion (the *incretin* effect)[1]. Consequently, GLP-1 has been intensely studied in relation to human diabetes. Endogenous GLP-1 has a plasma half-life of 1–2 min, due to a rapid degradation and inactivation by the enzyme dipeptidyl peptidase IV (DPP-IV), rendering natural GLP-1 impractical for diabetic treatment[1]. Exenatide is a synthetic GLP-1 mimetic and a potent GLP-1 receptor agonist with a half-life of 3–4 h after subcutaneous injection in humans due to DPP-IV resistance[2, 3]. GLP-1 mimetics increase insulin secretion, improve insulin sensitivity, reduce fasting blood glucose and decrease plasma glucagon levels in diabetic and non-diabetic human subjects[4–7]. Further, GLP-1 mimetics decrease body weight in obese, non-diabetic and diabetic human patients through increased satiety, reduced appetite and slowed gastric emptying[6–9]. Nausea and vomiting are the most common adverse reactions, especially during the initial weeks, and a stepwise dose escalation is recommended[6, 7]. Exenatide treatment may cause hypoglycemia if combined with other anti-hyperglycaemic treatments such as biguanides or sulfonylureas[10].

In cats, the prevalence of obesity is high, and as in humans, feline obesity increases the risk of several medical complications such as insulin resistance and feline diabetes mellitus[11–13]. Obese cats often maintain normal fasting blood glucose, presumably due to good control of hepatic endogenous glucose production compared with human prediabetes (impaired fasting glucose)[14]. However, feline obesity has been associated with insulin resistance, impaired glucose tolerance and an abnormal insulin response during a glucose tolerance test[12]. Further, the circulatory level of leptin was increased in obese compared to lean cats, and hyperleptinemia was linked to a decreased insulin sensitivity[11, 15]. In contrast, the association between obesity, adiponectin and insulin sensitivity is controversial in the cat[11, 16].

The number of studies investigating the effect of GLP-1 mimetics in cats is still limited. Single-dosing of exenatide increased insulin secretion in healthy, lean cats without adverse effects[17, 18]. In a recent study, treatment for seven consecutive days with a GLP-1 mimetic increased insulin secretion, decreased glucagon secretion and reduced body weight in healthy cats. Treatment was associated with anorexia, vomiting or diarrhea in the majority of cats, which may be explained by the use of a relatively large dose per kg bodyweight compared to human recommendations[19].

The pathophysiology of feline diabetes mellitus (FDM) is similar to human type 2 diabetes (T2D), including a strong association with obesity, insulin resistance and β-cell failure causing impaired insulin secretion[20].

Currently, insulin is the appropriate therapeutic option for feline diabetes, but may be inadequate for optimal clinical control in some cats and carries the risk of inducing hypoglycaemia[21, 22]. Based on the similarities between feline and human obesity and diabetes, it is possible that obese or diabetic cats could benefit from treatment with GLP-1 mimetics.

The aim of this study was to evaluate the effect and safety of long-term (12 weeks) exenatide treatment in healthy, spontaneously (not experimentally induced) obese client-owned cats. To the best of our knowledge, this is the first prospective double-blinded, placebo-controlled study with a GLP-1 mimetic in obese cats.

## Materials and Methods

### Animals, clinical examination, blood sampling and handling

Cats were enrolled in the study from March 2013 to May 2014. All were privately owned, recruited through local newspaper ads and owners provided written informed consent upon enrolment. The study was approved by the Danish National Committee on Animal Experimentation, the Danish Health and Medicines Authority and the Local Ethical and Administrative Committee at the Department of Veterinary Clinical and Animal Sciences, University of Copenhagen. All cats were indoor confined, above 3 years of age, had a body condition score (BCS) of at least 7 on a 9-point scale and were not receiving any medication. The medical history was obtained using a standardized questionnaire. After an overnight fast (10–12 h) all cats were subjected to a thorough clinical examination including measurement of body weight (BW) in kg (Kruuse MS-20, Kruuse, Langeskov, Denmark) and body condition scoring on a 9-point scale[23]. Health status was confirmed by urine and blood analyses including biochemistry, a complete blood count and thyroid profile. Blood samples were obtained by jugular venipuncture using a 21-gauge butterfly and samples were collected into two 4-mL serum (Greiner Bio-One, GmbH, Kremsmünster, Austria) and three 2-mL EDTA tubes (BD, Belliver Industrial Estate, Plymouth, United Kingdom). Serum tubes were left to clot at room temperature while EDTA tubes were immediately placed on ice. Samples were centrifuged within 30 min (EDTA) or after a minimum of 30 min (serum) at 3500 rpm for 10 min at 4°C and stored at -80°C for later analysis. A urine sample was collected by cystocentesis. Following blood sampling, all cats were fitted with two intravenous catheters, one in the cephalic vein (22-gauge) and one in the medial saphenous vein (20-gauge). None of the cats were sedated for the blood sampling or catheter placement. Afterwards cats had 3 h of undisturbed fasting cage rest, before an intravenous glucose tolerance test (IVGTT) was performed.

### Intravenous glucose tolerance test

The IVGTT followed a previously described protocol[16]. Following at least 3 h of cage rest, a glucose bolus (1 g/kg body weight) was injected through the cephalic catheter over 30 seconds. Through the other catheter, 2 mL samples of blood were collected into ice-chilled 2-mL EDTA tubes at times 0 (prior to injection), 2, 5, 10, 15, 30, 45, 60, 90 and 120 min after the glucose injection. Prior to each blood sampling, 0.5 mL of blood was drawn into a separate syringe to avoid collection of diluted catheter blood; this was re-injected immediately after collection of the actual sample. Catheter patency was maintained by flushing with 0.5 mL of heparinized saline. Blood samples were kept on ice after collection, centrifuged within 1 h at 3500 rpm for 10 min at 4°C and stored at -80°C for later analysis. After completion of the IVGTT, cats were hospitalized overnight, offered a meal and placed on intravenous fluids (Ringer Acetate, Fresenius Kabi, Uppsala, Sweden) for 8 h to replenish circulatory volume. Food was withheld after 10 PM.

### Dual-energy x-ray absorptiometry

On the following day, cats were shortly anaesthetized for a dual-energy x-ray absorptiometry (DXA) scan. Cats were given 0.2 mg/kg methadone (Comfortan Vet, Dechra Veterinary Products, Uldum, Denmark) intramuscularly followed by 0.2–0.4 mg/kg diazepam (Stesolid emulsion, Actavis, Gentofte, Denmark) intravenously. Anesthesia was induced and maintained by injection of propofol (Rapinovet, Shering Plough Animal Health, Ballerup, Denmark) intravenously; 6 mg/kg for induction and approximately 15 mg/kg for maintenance depending on individual need. The cat was placed in sternal recumbence with the front legs extended cranially and the hind legs extended caudally for the DXA scan. Scans were performed with Lunar Prodigy Advance (GE Medical System Lunar, Madison, WI, USA), and the body composition assessed by the accompanying software (Lunar Prodigy enCORE 2011, version 13.60.033, GE Medical System Lunar, Madison, WI, USA).

### Study design and treatment protocol

The study was a randomized, double-blinded, placebo-controlled, parallel group study. All cats were enrolled within a period of 14 months. Following the procedures described above, cats were randomized to 12 weeks of either placebo (saline) or exenatide treatment (Byetta, Eli Lilly Denmark, Herlev, Denmark). A block size of six was used for randomization, and if any cat dropped out within the initial 28 days of participation, the next cat was allocated blindly to the same treatment arm. Completion of at least 28 days of the study period was required for the cat to be included in the final data analysis. Exenatide was diluted 1:10 following a protocol provided by the manufacturer using the same excipients as included in the original Byetta formulation. After manufacturing, the dilution was stored at room temperature for a maximum of 30 days in agreement with the recommendations for Byetta[24]. Exenatide was administered subcutaneously at a dose of 0.5 μg/kg BW twice daily for the first 4 weeks increasing to 1.0 μg/kg BW twice daily from week 5 to 12 to mimic the treatment protocol recommended in humans. Dosage was based on the preliminary results of a recently published feline study[25]. To preserve blinding, the placebo and exenatide were given in identical volumes (0.02 ml/kg body weight for the initial 4 weeks). Both liquids were clear and colorless. Owners received oral and written instructions regarding the treatment protocol, and performed a supervised saline injection to confirm a correct injection technique. The cats were not fed a standardized diet, instead owners were asked to refrain from making any dietary changes for their cat and to continue feeding the exact diet as before enrolling in the study. Further, owners were instructed to contact the hospital at any signs of an adverse reaction or change in their cat’s demeanor. A standardized diary was used by all owners for logging of any adverse reactions or missing injections. Cats were reexamined after 2, 4, 8 and 12 weeks of treatment. Prior to each re-check the cats were fasted for 12 h, and each visit included a clinical examination and assessment of BW and BCS. A standardized questionnaire was used to obtain the latest medical history. Prior to each visit owners were asked to rate their cats appetite on a 5-point scale, where 1 = the cat is always hungry, 3 = the cats eats when food is offered but appears satiated between feedings, and 5 = the cat shows no interest in food. Urine and blood samples (for biochemistry, complete blood count and feline pancreatic lipase immunoreactivity) were obtained as described previously and analysed within 24 h for assessment of any adverse reactions. At the final visit (week 12) the cats were subjected to a follow-up IVGTT and DXA scan following the same protocol as used at the time of inclusion. At that time all treatments were discontinued.

### Study end points

The primary endpoint was change in insulin concentration during the IVGTT at study initiation and end of treatment (week 12 or last observation carried forward). Secondary endpoints included changes in glucose concentration and glucose tolerance parameters, glucagon concentration and BW as well as an evaluation of long-term safety.

### Glucose, hormone and adipokine analyses

#### Glucose and insulin analyses

Samples were analysed collectively after all cats had completed the study. Glucose concentrations were measured by a Glucose Hexokinase II method (Advia 1800, Siemens Healthcare Diagnostics Inc., Tarrytown, NY, USA). Plasma insulin concentrations were determined by a radioimmunoassay (INS-IRMA, DIAsource, Nivelles, Belgium) by the laboratory where this assay was previously validated in cats[26]. The glucose half-life (T_½_) and disappearance of glucose (K_glucose_) were based on a linear regression of log_10_ of the glucose concentrations between 15 and 90 min during the IVGTTs and calculated as:

$$K_{glucose}\;=\;\frac{|b|\times 100}{{log}_{10}e}$$

and

$$T_{½}\;=\;\frac{{log}_{e}2\times 100}{K_{glucose}}$$

where |b| is the absolute value of the slope and e is the natural logarithm base[27].

#### Glucagon, total GLP-1 and exenatide analyses

Fasting plasma glucagon levels were determined at week 0 and 12 using a sandwich ELISA method (Glucagon ELISA, Mercodia, Uppsala, Sweden) previously validated in cats; the detection limit was 1 pM and CV < 10%[19, 28]. For the measurement of fasting total GLP-1, we used a well-characterized in-house developed radioimmunoassay (codename 390) measuring total GLP-1 levels as previously described; the detection limit was 3pM and CV < 10%[29]. Fasting plasma exenatide was measured using an in-house developed radioimmunoassay (codename 3145) with CV < 6% and a detection limit of 0.5 pmol/L[30].

#### Leptin and total adiponectin analysis

Fasting plasma leptin was determined by radioimmunoassay (Multi-species Leptin RIA, Merck Millipore, Darmstadt, Germany) previously validated in cats[31]. Total serum adiponectin concentration was measured by a commercially available human adiponectin ELISA following the manufacturer’s guideline for analysis of feline samples (Human Adiponectin ELISA, High Sensitivity; BioVendor—Laboratorni medicina, Brno, Czech Republic). This assay was previously validated in cats in our laboratory and by Tvarijonaviciute and colleagues [16, 32]. All samples were measured in duplicate using a single microplate and the CV for each pair of samples was <7.4%.

### Statistical analysis

Group characteristics at baseline were assessed using the unpaired t-test (BW, body fat percentage and age) and Fisher’s Exact Test (gender). The area under the curve for glucose and insulin (AUC_glucose_ and AUC_insulin_) during the IVGTT was calculated from all serial measurements from 0 to 120 minutes by the trapezoidal method (GraphPad Software Inc, La Jolla, CA). Most variables were measured at week 0 and 12, and the change in each cat was calculated as results obtained in week 12 minus the results obtained in week 0. Treatment effect was evaluated by comparing the changes between treatment groups using either the t-test or the Kruskal-Wallis test as appropriate (SAS, version 9.4; SAS Institute Inc, Cary, NC). Data distribution was assessed using the Shapiro-Wilk test with a significance level set at 0.05 (IBM SPSS Statistics, version 22; IBM Corporation, Armonk, New York). For variables measured at more than two time points, comparisons between groups were performed by mixed-ANOVA. Results are presented as median and range unless otherwise stated. Statistical significance was set at P < 0.05, while a P value ≤ 0.1 was considered a trend.

## Results

### Included animals

A total of 12 healthy cats completed the study. Fourteen were originally enrolled, but two cats dropped out before day seven due to aggression towards the owners during injections, and per protocol these cats were not included in any of the statistical analyses and will not be further discussed. Another two cats (one in each treatment group) were withdrawn after 4 and 8 weeks respectively for the same reason. Both cats completed an IVGTT at the time of withdrawal and the results are included in the final analysis. For all other variables, these two cats are included in the calculations for as long as they were included in the study. Thus, a total of six cats in each treatment group were included in the final analysis. Median age at time of inclusion was 86.5 months (range 63–119 months) in the exenatide treated group (EX) and 91.0 months (range 66–146 months) in the placebo group (PL) (P>0.05). Eight cats were castrated males (EX = 5, PL = 3), four were spayed females (EX = 1, PL = 3) and the gender distribution was not significantly different between treatment groups (P>0.05). Included breeds were Domestic Short Hair (EX = 4, PL = 1), British Short Hair (EX = 2, PL = 1), Burmilla (PL = 2) and mixed breeds (PL = 2). The median body weight at time of inclusion was 8.19 kg (range 6.65–10.8 kg) in EX and 6.64 kg (range 5.04–10.27 kg) in PL, P = 0.17. Median body fat percentage at baseline was 45.5% in EX (range 32.6–60.2%) and 46.3% in PL (range 41.0–61.1%), P = 0.54.

### Exenatide concentration

Fasting, plasma exenatide concentration was measured at week 4 and 12 approximately 2–3 h after the latest injection. The median exenatide concentration in EX was 50 pM (range 20–150 pM) at week 4 and 70 pM (range 20–410 pM) at week 12. In PL the median concentration was 0 pM (range 0–3 pM) at week 4 and 0 pM (range 0–2 pM) at week 12. The exenatide concentration was significantly higher in EX compared to PL at both week 4 and 12 (P = 0.03 and < 0.01, respectively). The owners were unaware of this analysis being performed.

### Insulin secretion, glucose concentration and glucose tolerance

**Fig 1:**
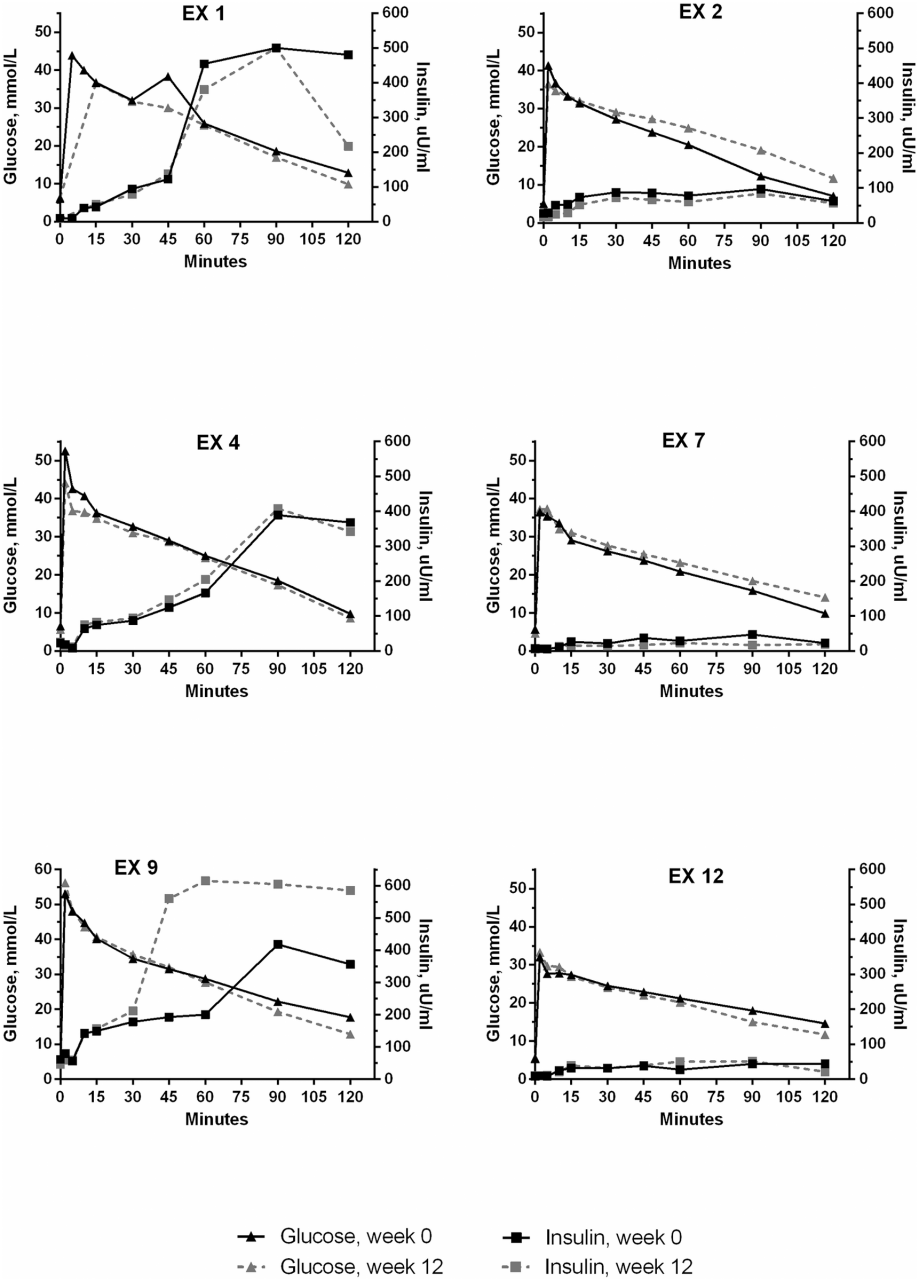
Plasma insulin and glucose concentration in exenatide treated cats. Plasma insulin and glucose concentration during an intravenous glucose tolerance test in 6 obese client-owned cats before and after 12 weeks of twice-daily subcutaneous exenatide treatment. Insulin and glucose were measured immediately before and for 120 min following a 1 g/kg intravenous bolus of glucose.

**Fig 2:**
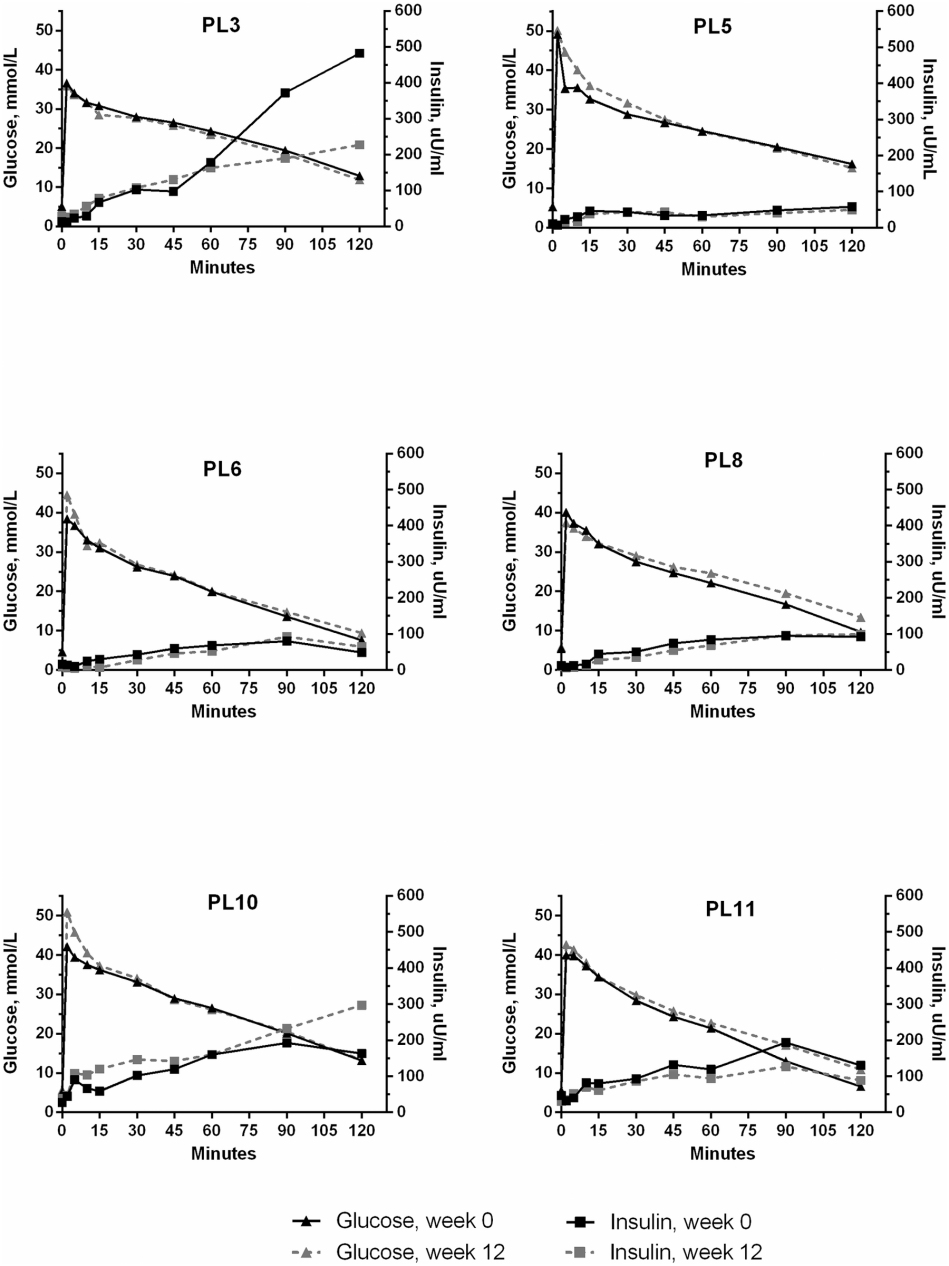
Plasma insulin and glucose concentration in placebo treated cats. Plasma insulin and glucose concentration during an intravenous glucose tolerance test in 6 obese client-owned cats before and after 12 weeks of twice-daily subcutaneous placebo treatment. Insulin and glucose were measured immediately before and for 120 min following a 1 g/kg intravenous bolus of glucose.

**Fig 3:**
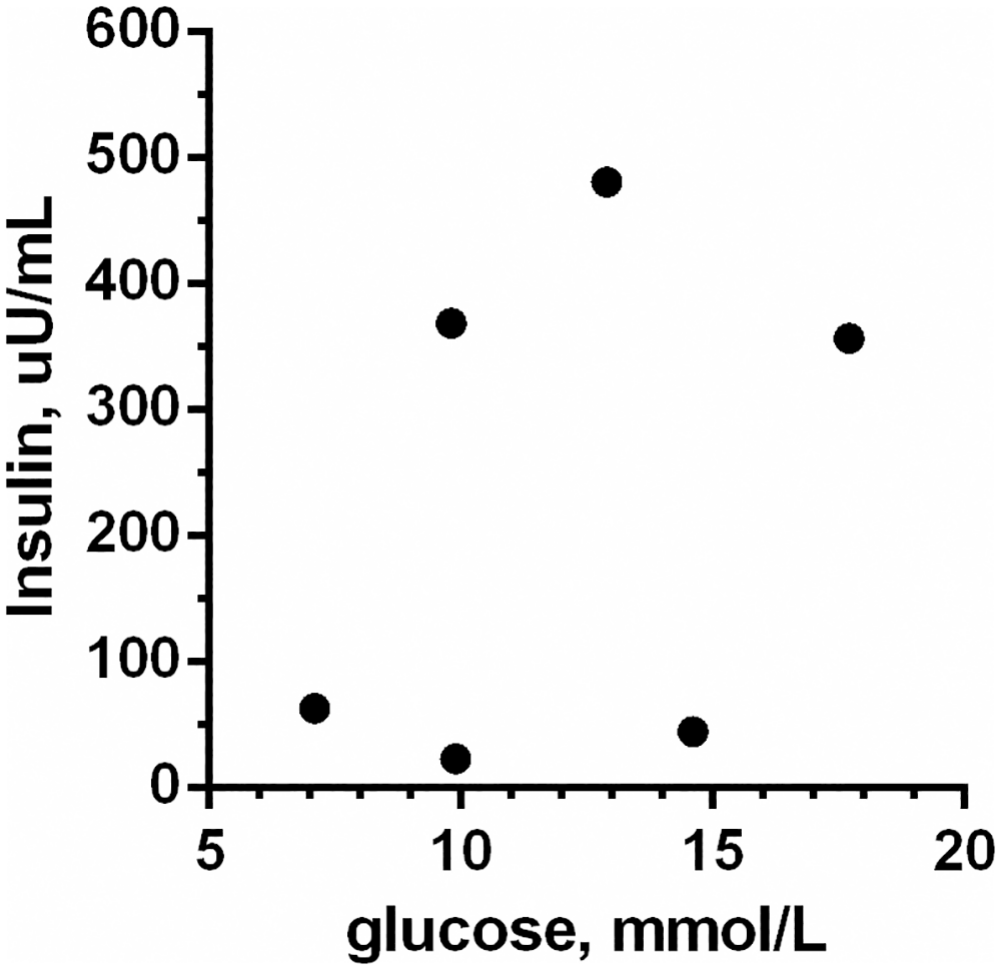
Individual differences in plasma insulin and glucose levels 120 minutes after a glucose bolus. Plasma insulin and glucose concentration 120 min after a 1 g/kg glucose bolus in six obese client owned cats randomized to the exenatide treatment group. Measurements were obtained before treatment initiation.

Median and range values for the insulin and glucose measurements are presented in Table 1. Exenatide treatment did not change baseline plasma glucose or baseline insulin concentration. The AUC_insulin_ was calculated for the early response (0 to 30 min), the late response (30 to 120 min) and for the entire 120 min period. Exenatide did not have a significant effect on any of the AUC_insulin_ results. The individual insulin secretion and corresponding glucose concentration curves during the IVGTT at week 0 and week 12 are shown for the EX and PL group in Figs 1 and 2 respectively. In the EX group there was a discernable individual variation in the insulin concentrations, which is also evident from a diagram showing the glucose and insulin concentration 120 min after the glucose bolus for EX at week 0 (Fig 3). AUC_glucose_ was calculated for the entire 120 min period and was not different between EX and PL. Finally, exenatide did not affect the indirect measurements of glucose tolerance: K_glu_ or T_½_ (Table 1).

**Table 1:** Hormone, glucose and body composition analyses before and after 12 weeks of either exenatide or placebo treatment.

Parameter	Unit	Exenatide, week 0	Exenatide, week 12	Placebo, week 0	Placebo, week 12	p-value
Baseline insulin	μU/mL	16.4 (7.1–61.7)	13.7 (6.2–46.0)	14.9 (11.0–46.6)	19.3 (8.9–38.5)	0.52
AUC insulin 0–120	μU/ml/min	17405 (3669–36341)	17274 (2080–55032)	11963 (4988–26360)	9557 (4498–21671)	0.64
AUC insulin 0–30	μU/ml/min	1608 (529–4023)	1282 (382–4282)	1395 (836–2199)	1405 (386–3412)	0.82
AUC insulin 30–120	μU/ml/min	15512 (3140–34950)	15582 (1698–50750)	10366 (3917–24640)	8256 (3623–18260)	0.65
Baseline glucose	mmol/L	5.6 (5.1–6.6)	5.2 (4.6–6.4)	5.3 (4.7–5.5)	5.4 (4.5–5.8)	0.17
AUC glucose 0–120	mmol/L/min	2811 (2437–3524)	2838 (2396–3374)	2759 (2444–3146)	2868 (2558–3234)	0.30
K-glucose	%/min	0.86 (0.55–1.23)	0.85 (0.68–1.00)	0.82 (0.60–1.26)	0.79 (0.59–1.03)	0.76
Glucose T½	min	81.1 (56.2–127.0)	82.8 (69.2–102.6)	85.0 (55.0–114.9)	87.6 (67.2–117.5)	0.77
Glukagon, fasting	pM	11.5 (1.9–17.7)	2.6 (0.7–4.9)	9.8 (3.6–14.4)	7.3 (3.2–14.4)	0.25
Total GLP-1, fasting	pM	6.9 (4.9–8.2)	6.7 (4.7–7.9)	8.8 (5.9–11.3)	9.0 (6.5–10.0)	0.20
Percent weight loss*	%		5.1 (1.7–8.4)		3.2 (-5.3–5.7)	0.10
% Body fat	%	45.5 (32.6–60.2)	38.1 (25.1–57.7)	46.3 (41.0–61.1)	41.2 (29.1–56.8)	0.97
Total fat	g	3437.3 (2083.9–6286.5)	3446.3 (1479.1–5725.2)	3127.7 (1996.9–4366.7)	2585.2 (1813.2–4021.4)	0.89
Adiponectin, fasting	μg/ml	0.66 (0.43–0.82)	0.53 (0.39–0.86)	0.78 (0.47–0.92)	0.85 (0.38–0.90)	NS
Leptin, fasting	ng/ml	42 (23–48)	42 (10–47)	38 (25–43)	43 (20–51)	0.28

Hormone concentrations, glucose tolerance estimates and body composition in obese client-owned cats before and after 12 weeks of treatment with either exenatide (n = 6) or placebo (n = 6). Values are presented as median and range. The p-value is obtained by comparing the observed changes (result from week 12 minus result from week 0) between the two treatment groups.

Abbreviations: AUC, area under curve; K-glucose, disappearance coefficient for glucose; Glucose T½, half-life for glucose in plasma; GLP-1, glucagon-like peptide-1.

*Calculated as weight loss in percentage of baseline, i.e. (body weight week0-week12)/body weight week0.

### Fasting glucagon and total GLP-1 concentration

**Fig 4:**
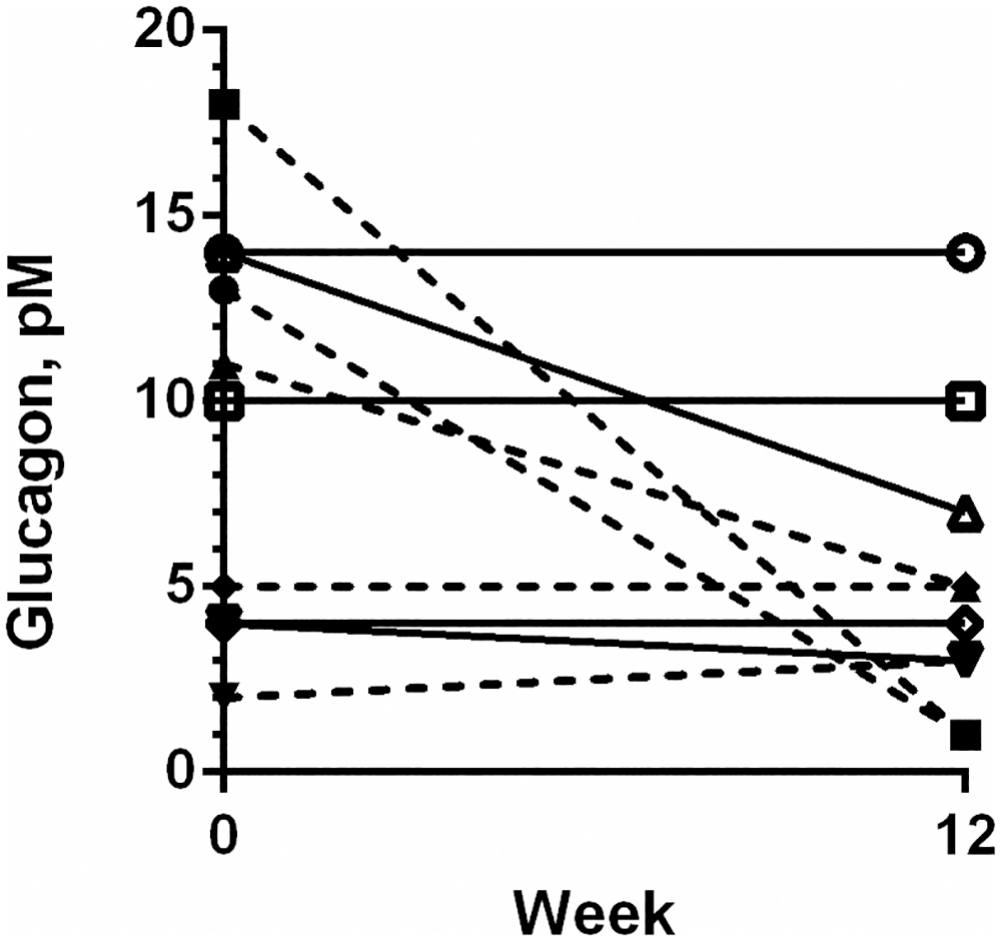
Fasting plasma glucagon concentration. Fasting, plasma glucagon concentration in ten obese client-owned cats before and after 12 weeks of exenatide (broken lines, n = 5) or placebo (solid lines, n = 5) treatment. P = 0.25.

Glucagon and total GLP-1 were only measured in five cats from each group due to insufficient sample volume in two cats. Based on visual evaluation of the data, the fasting glucagon concentration decreased markedly in several EX cats, especially in cats with a higher glucagon concentration at baseline, whereas it was unchanged in all PL cats. However, this did not reach statistical significance (Fig 4 and Table 1, P = 0.25). Total GLP-1 concentration was unaffected by exenatide treatment (Table 1).

### Body weight, body composition and adipokines

**Fig 5:**
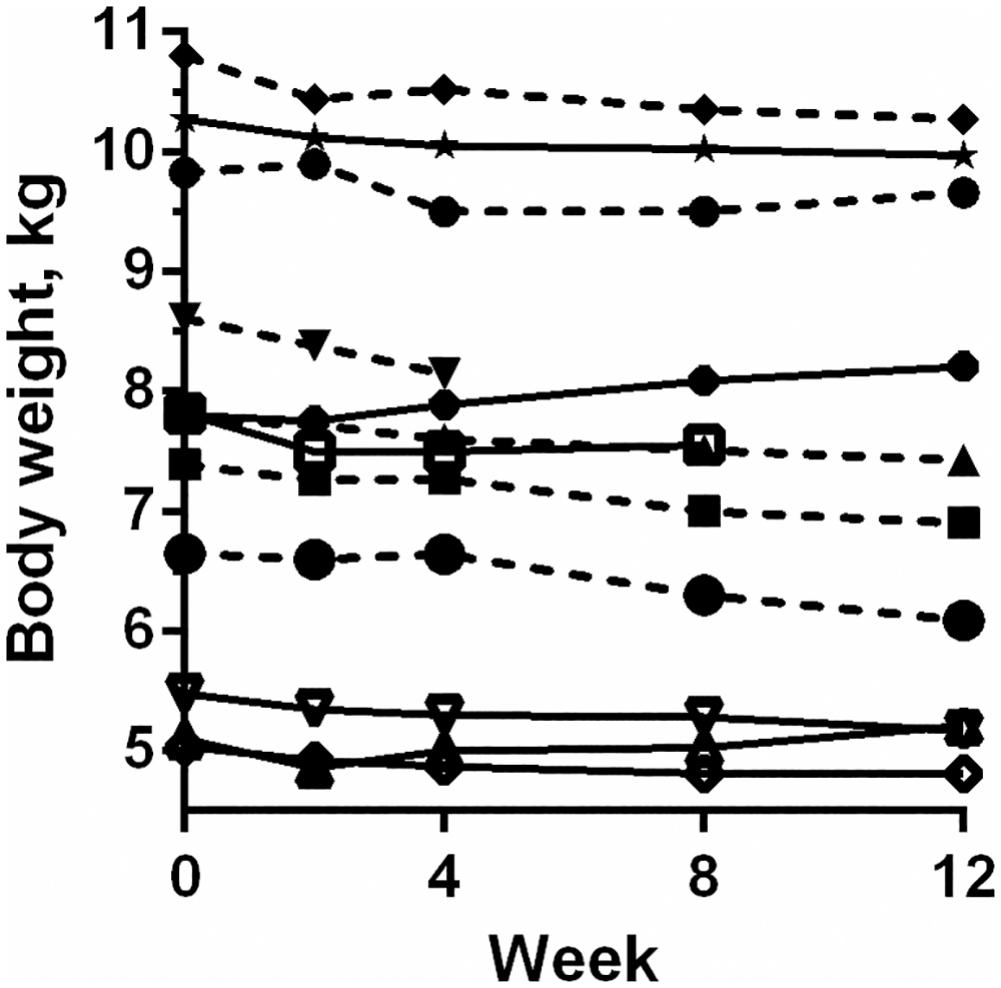
Body weight before, during and after the study period. Body weight of 12 obese client-owned cats before and after 12 weeks of treatment with either exenatide (broken lines, n = 6) or placebo (solid line, n = 6). Two cats (one in each group) were withdrawn after 4 and 8 weeks respectively.

All cats in EX lost weight (Fig 5), and overall there was a significant absolute weight loss in this group (P < 0.001). In PL, four cats lost weight while two cats gained weight (Fig 5), and overall the BW was not significantly changed in this group (P = 0.45). For comparing the weight loss between the exenatide and placebo treated groups, the relative weight loss in each group was calculated, i.e. weight loss as percentage of baseline BW (Table 1). There was a trend for a greater relative weight loss in EX compared to PL (P = 0.10). Exenatide did not change total body fat percentage or total amount of body fat compared to placebo. Finally, exenatide did not affect plasma leptin or total adiponectin concentrations.

### Safety and compliance

For the 12-week period, the median number of total reported missed injections per cat in EX was 1 (range 0–10) and 5 in PL (range 0–8). All owners returned used syringes and empty or expired drug vials, indicating that the medication had been administered as instructed. As some utensils were discarded at home, the counting of the returned items was not used as a quantitative measure of compliance.

The medical histories obtained at the time of inclusion showed that several cats had single bouts of vomiting on an irregular basis (primarily hair balls). The frequency of this did not change for the affected cats during this study in either group. In EX there were three reported incidents of adverse reactions, all of which occurred after 3 to 4 weeks of treatment. One cat vomited repeatedly for 2 days resolving spontaneously, and general appetite and mood were unaffected. Another cat had self-limiting diarrhea for 1 day that coincided with an abrupt diet change (this was a client violation of protocol, and the original diet was immediately resumed). A third cat was suspected of a hypoglycemic incidence characterized by fatigue, slight head bobbing and blood glucose of 3.5 mmol/L. The cat was treated at home by feeding and recovered without further incidents.

Blood and urine samples collected at time of inclusion and study end (Table 2) were stable with no signs of adverse reactions. Blood samples collected at rechecks were unremarkable except from an increase in the acute phase protein serum amyloid A in two cases (66.8 mg/L and 68.9 mg/L respectively, reference range 0.0–5.0 mg/L). One was a PL cat without any clinical symptoms, and the other was the EX cat with diarrhea and was detected the day after the incidence of diarrhea. Both cases were resolved at the following blood re-check. Several cats in both groups had occasional reactions to the injections (vocalizing or trying to get away). A total of four cats out of the 14 cats that were initially recruited did not complete the total 12 weeks of study due to aggressive behavior in relation to the injections (EX = 3, PL = 1). Two of these (both from EX) completed less than one week of the study and were not included in the data analysis, as previously mentioned. All cats were thoroughly examined at every re-check and no signs of cutaneous reactions were discovered.

**Table 2:** Biochemical blood analyses and hemograms before and after 12 weeks of treatment with either exenatide or placebo.

	Exenatide (n = 6)		Placebo (n = 6)		Reference range
	Week 0	Week 12	Week 0	Week 12	
WBC	6.9 (5.6–8.5)	6.2 (5.6–6.9)	7.5 (4.9–8.9)	6.1 (5.1–13.9)	5.5–19.5 10^9^/L
PCV	42.3 (34.8–45.6)	41.7 (30.2–46.0)	39.0 (37.5–45.5)	39.0 (37.2–46.0)	24.0–45.0%
ALT	59.0 (30.0–63.0)	49.0 (31.0–73.0)	53.5 (42.0–61.0)	48.5 (43.0–57.0)	6.0–84.0 U/L
ALP	53.5 (22.0–130.0)	45.0 (26.0–133.0)	70.0 (24.0–87.0)	56.5 (26.0–77.0)	25.2–96.0 U/L
GGT	0.0 (0.0–0.0)	0.0 (0.0–1.0)	0.0 (0.0–1.0)	0.0 (0.0–0.0)	0.0–5.4 U/L
BUN	9.2 (6.9–11.7)	8.9 (6.9–10.6)	8.0 (7.0–10.0)	8.4 (7.4–10.4)	6.7–10.0 mmol/L
Creatinine	126.5 (109.0–156.0)	143.0 (123.0–154.0)	98.0 (76.0–142.0)	101.0 (79.0–152.0)	60.0–170.0 μmol/L
Cholesterol	4.19 (2.88–8.66)	5.17 (3.34–8.24)	4.66 (3.11–6.26)	4.35 (2.84–6.53)	2.46–3.37 mmol/L
Bilirubin	0.8 (0.3–1.5)	1.0 (0.7–1.5)	0.8 (0.5–5.1)	0.6 (0.0–3.3)	0.0–3.4 μmol/L
Albumin	37.2 (35.6–40.4)	38.1 (34.1–41.0)	36.3 (33.6–40.5)	35.3 (33.0–39.2)	28.0–40.0 g/L
Protein	70.2 (65.9–75.5)	70.9 (63.2–79.8)	77.7 (75.5–79.7)	73.2 (70.7–81.8)	54.0–74.0 g/L
Fructosamine	326.0 (295.0–354.0)	333.5 (309.0–369.0)	360.5 (312.0–403.0)	331.5 (312.0–390.0)	221.0–341.0 μmol/L
Bile Acids	1.0 (0.0–3.0)	1.0 (1.0–6.0)	1.0 (1.0–11.0)	1.0 (0.0–1.0)	1.0–5.0 μmol/L
SAA	0.0 (0.0–0.1)	0.2 (0.0–0.4)	0.1 (0.1–0.4)	0.0 (0.0–0.2)	0.0–5.0 mg/L
fPLI	negative (n = 6)	negative (n = 6)	negative (n = 6)	negative (n = 6)	negative/positive

Fasting blood parameters measured at time of inclusion and after 12 weeks of either exenatide or placebo treatment. Values are represented as median and range. The reference range from our laboratory is also listed.

Abbreviations: WBC, white blood cells; PCV, packed cell volume; ALT, alanine aminotransferase; ALP, alkaline phosphatase; GGT, gamma-glutamyl transpeptidase; BUN, blood urea nitrogen; SAA, serum amyloid A; fPL, feline pancreas-specific lipase.

The exact daily food intake was not measured. Instead, the owners rated their cat’s appetite on a pre-defined 5-point scale, where 1 indicated that the cat was always hungry or begging for food and 5 indicated that the cat was not interested in food. According to the owners, appetite decreased by a change of 1–3 points in five of the six cats in EX and remained unchanged in one cat. In PL, appetite was decreased by a change of 2 points in one cat, remained unchanged in two cats and increased by a change of 1–2 points in three cats. No cases of anorexia or reluctance to eat were reported.

## Discussion

To the best of our knowledge this is the first placebo controlled, long-term study evaluating the effect of exenatide in naturally obese, client-owned cats. The main findings of this study were: (i) the insulin potentiating effect of chronic exenatide treatment in obese cats varied among individuals, and appeared less pronounced compared to previous studies in purpose-bred lean cats, (ii) there was a trend for a weight loss effect in exenatide treated obese cats even without dietary changes, (iii) 12 weeks of treatment with exenatide was well tolerated in obese, but otherwise healthy, cats.

Currently, GLP-1 mimetics have only been studied in healthy and predominantly lean cats, and direct comparisons between studies are impeded by the variation in methods and dosage protocols used[17–19, 25, 33]. In this study, 12 weeks of twice daily exenatide treatment did not increase the total insulin secretion (AUC_insulin_) during an IVGTT compared to placebo. This is in contrast to previous studies in lean cats, where insulin was notably increased by exenatide[17, 18, 25]. The IVGTT was chosen for this study, because the cats were client-owned, and in our experience this test is well tolerated and induces a minimum of stress[16]. It is possible that a meal response test causing an incretin effect or a hyperglycemic clamp could have yielded a different outcome.

Despite not detecting an overall increase in insulin release, this study did find a marked individual variation in the magnitude of the insulin response to exenatide. This may be caused by the obese, client-owned cats being more heterogeneous at baseline compared to previously studied purpose-bred populations. Indeed, at week 0, the AUC_insulin_ in EX varied almost 10-fold. A high insulin concentration most likely results from insulin hypersecretion in order to compensate for peripheral insulin resistance. Also, consistent with previous studies in obese cats, the insulin responses to i.v. glucose were delayed, despite immediate and marked glucose increments[12, 34]. This suggests a reduced glucose sensitivity of the pancreatic beta-cells in the obese cats. The heterogeneity observed in the insulin secretion patterns at baseline in this study is consistent with the notion that not all obese cats become insulin resistant[12]. A marked heterogeneity would make it difficult to find a statistical difference, and may partly explain the inconsistent effect of exenatide on insulin secretion in this study. In future studies, an initial assessment of insulin resistance and glucose tolerance should precede the randomization process to fully elucidate the effect of exenatide on insulin secretion in obese cats.

The insulin augmentation produced by exenatide was negligible in five of six cats. This is in contrast to previous feline studies using the same dosage but for a shorter period of time, where total AUC_insulin_ increased with approximately 350% [18, 25]. Our study could indicate that in obese cats, the effect of exenatide may be less pronounced. Despite a large inter-individual variation in AUC_insulin_ in our population, our sample size had a power of 99% to detect a 200% increase in AUC_insulin_ at a 5% significance level based on a post-hoc calculation (using the mean and SD from our study: 15679±11352). This calculation cannot predict the true exenatide effect; but in combination with the results of this study, it does suggest that the effect of exenatide in obese cats is less than observed in lean cats regardless of the level of insulin resistance. One possible explanation could be the presence of GLP-1 resistance in obese cats. Resistance to GLP-1 is controversial in humans, but it has been suggested that the pancreatic β-cells may have a reduced ability to respond to incretins in some obese individuals[35]. Whether a similar condition exists in obese cats is unknown. Also, we did not test for the presence of anti-exenatide antibodies in our group of cats. In humans, the transient development of anti-exenatide antibodies is well described, peaking in the treatment weeks 6 to 16[36]. However, most exhibit a low antibody titer, which apparently does not affect the clinical efficacy of exenatide[36].

One limitation of this study is that the timing of the last exenatide injections in relation to the initiation of the final IVGTT was not standardized, and this may have affected the insulin secretion. A previous report suggested that the insulinotropic effect of exenatide lasts approximately 120 min following a single subcutaneous injection in cats[18]. Still, following a chronic exenatide administration, a general improvement in β-cell secretory function could be expected, which should not be affected by the timing of the latest injection[37].

Consistent with a previous study, there was a significant absolute weight loss in the EX group[18]. However, the previous study did not include a control group, and in our study the weight loss was no longer statistically significant when compared to the placebo treated group. In our study, there was only a tendency for exenatide to increase the relative weight loss compared to placebo, although a weight loss was observed in all EX cats whereas a weight gain was observed in some PL cats. We still believe it is an important finding, especially because it occurred without dietary modifications, and an insufficient sample size may explain the lack of statistical significance. The rate of weight loss was compatible with what is considered safe in cats, in contrast to the weight loss rate noted in lean, purpose-bred cats in a previous study[18, 38]. The clinical impact of the weight loss rate observed in our study may be small, and future long-term studies are needed to fully evaluate this.

In humans, GLP-1 analogues are believed to cause a weight loss by reducing appetite and slowing gastric emptying[6]. Whether similar mechanisms occur in cats is not fully understood. In our study, the total daily food intake was not measured directly, however, the majority of EX cat owners believed their cat’s appetite had decreased during the study. This may partly explain the weight loss in this group, and is consistent with findings in lean cats[19]. Importantly, no cats became anorexic, and the appetite reduction was generally described as cats that were no longer constantly begging for food, but rather seemed satiated between meals. Body weight was recorded in seven cats (three EX, four PL) 4 weeks after study completion, and all three cats from EX had gained weight (range 0.15–0.41 kg), and the owners reported an increase in appetite after discontinuation of exenatide. None of the PL cats had gained weight after finishing the study. This further supports the notion of exenatide reducing both appetite and BW in obese cats, although further studies using a controlled feeding and registration of appetite and food consumption is needed to fully confirm this theory. Exenatide did not change the body composition of obese cats, consistent with studies of similar duration in obese humans, where a significant weight loss was unrelated to a change in body composition[7, 8]. Fasting levels of the adipokines leptin and adiponectin were likewise unaffected by exenatide. Leptin concentration is highly correlated with fat mass in cats, while the association between obesity and adiponectin in cats is debated [11, 16, 31, 39]. Hence, the unchanged body composition likely explains the lack of change in leptin and possibly adiponectin.

Hyperglucagonemia has been observed in obese humans with normal glucose tolerance and in T2D [28, 35]. Few reports exist concerning glucagon levels in obese cats, but hyperglucagonemia has been shown in diabetic cats in an older study[40]. Insulin has traditionally been the focus of attention for diabetic research, but in recent years glucagon has received increased attention, as hyperglucagonemia contributes to the hyperglycemia present in T2D. One area of interest is the suppression of glucagon secretion, which is one of the effects of GLP-1 mimetics in both T2D and in lean healthy cats[19, 33, 41–43]. In our study, fasting glucagon decreased in several exenatide treated cats. Glucagon may be affected by storage of the samples, but in a recent human study it was shown that the decline in glucagon concentration primarily occurred in the initial two weeks of sample storage, and that following this time-period the reduction was comparable between individual samples[29]. In our study, all samples were analysed collectively following 8–15 months of storage, and thus we do not believe that glucagon degradation is the cause for the observed reduction in glucagon concentrations. The reduction in fasting glucagon concentration was not statistically significant, but this may well be due to a lack of power, since only 10 cats (five EX, five PL) were included in this analysis due to insufficient sample volume. Our results suggest that exenatide may have a glucagonostatic effect in obese cats, but further studies measuring glucagon concentration during a glucose tolerance test in a larger population are needed to confirm this theory.

Exenatide was well tolerated in the obese cats and adverse effects were mild and self-limiting. The adverse reactions observed in the present study are consistent with the most common side effects typically reported in humans and cats[6, 7, 25, 33]. There was one case of diarrhea, which coincided with an abrupt change in the cat’s diet, and whether the diet or exenatide caused the diarrhea is unknown. There was an intermittent, vocal reaction to the injections in both treatment groups, and a total of four out of 14 owners (three EX and one PL) withdrew their cat from the study because they were unable to continue the injections at home. We cannot exclude that the exenatide formulation irritates at injection; it has not previously been reported as a problem but could be related to the dilution protocol. However, the vocal reactions were neither consistent nor limited to the EX, and could perhaps be caused by a lack of owner technique or confidence. No cutaneous reactions were found at any time.

Feline pancreatic lipase immunoreactivity was analysed at every recheck in our study, as some case reports have suggested that exenatide may increase the risk of pancreatitis in humans[44]. However, none of the cats had any clinical or biochemical evidence of pancreatitis, consistent with the results from a recent meta-analysis where treatment with GLP-1 mimetics was not associated with an increased risk of pancreatitis in humans[45]. One of the main challenges in this study is the evaluation of compliance. Used syringes and medicine bottles were collected from all participating owners and indicated a high compliance. However, some of the utensils were discarded at home by the owner and were not returned. On these few occasions we had to rely on the owners’ word for evaluation of compliance. Plasma exenatide concentration was measured without informing owners and strongly indicated that the medication was being administered in all EX cats. Individual variation in absorption as well as differences in the timespan between injection and blood sampling are presumably the causes for the variations observed in absolute concentration within the EX group[3]. In a previous study, plasma exenatide was only elevated above baseline for 90 min following a subcutaneous injection in cats[17]. Therefore the exenatide measurements do not guarantee that cats were also treated in the weeks between analyses. However, since the owners were unaware of this analysis being performed, there is no reason to believe injections were only administered on the days of the visits. All owners had volunteered and remained in frequent contact with the primary investigator throughout and beyond the 12-week study period, and overall compliance with regards to administration of the treatments was believed to be high. Apart from the initial health assessment of their cat, the owners did not receive any financial compensation for their participation in the study, and we believe our study represents a realistic setting for the clinical application of exenatide in obese client-owned cats.

Owners were instructed not to change their cat’s daily feeding routine, but based on verbal reports this was not strictly adhered to, and it was impossible to control the type of diet and amount of treats given. Also, it is possible that increased motivation after signing on for a clinical study may have led to the instigation of an unintentional weight loss regimen because some owners became more aware of their feeding habits. Although the feeding of various diets is likely a true representation of the clinical reality, the use of a standardized diet may prove a relevant amendment for future study protocols.

In conclusion, 12 weeks of treatment with exenatide was well-tolerated in obese cats, a potential veterinary target population for treatment with GLP-1 mimetics. Exenatide tended to decrease BW at an appropriate rate even without dietary changes, but did not alter body composition or adipokine levels. The insulin augmenting effect of exenatide was less pronounced in obese compared to lean cats, which may partly be explained by heterogeneity in the obese cats at baseline. The interaction between baseline insulin resistance and effect of exenatide in obese cats, as well as the effect on weight loss and appetite requires further investigations. Finally, exenatide may suppress fasting glucagon concentration in obese cats, which could be an important finding with regards to treatment of FDM, although this requires further research.

## Supporting Information

S1 Table Signalement of included cats. Signalement at the time of inclusion of the 12 cats included in the final data analysis. Abbreviations: MC, castrated male; FS, spayed female.
(XLSX)

S2 Table Owner evaluation of the cats’ appetite. The owners rated their cat’s appetite on a pre-defined 5-point scale before and after treatment with either exenatide or placebo. A score of 1 indicated that the cat was always hungry or begging for food. A score of 3 indicated that the cat was readily eating when food was offered but seemed satiated between meals. A score of 5 indicated that the cat was not interested in food.
(XLSX)

## References

[1] Holst JJ. The physiology of glucagon-like peptide 1. Physiol Rev 2007;87(4):1409–39. 17928588 10.1152/physrev.00034.2006

[2] Goke R, Fehmann HC, Linn T, Schmidt H, Krause M, Eng J, et al Exendin-4 is a high potency agonist and truncated exendin-(9–39)-amide an antagonist at the glucagon-like peptide 1-(7–36)-amide receptor of insulin-secreting beta-cells. J Biol Chem 1993;268(26):19650–5. 8396143

[3] Kolterman OG, Kim DD, Shen L, Ruggles JA, Nielsen LL, Fineman MS, et al Pharmacokinetics, pharmacodynamics, and safety of exenatide in patients with type 2 diabetes mellitus. Am J Health System Pharm 2005;62(2):173–81. 15700891 10.1093/ajhp/62.2.173

[4] Moretto TJ, Milton DR, Ridge TD, Macconell LA, Okerson T, Wolka AM, et al Efficacy and tolerability of exenatide monotherapy over 24 weeks in antidiabetic drug-naive patients with type 2 diabetes: a randomized, double-blind, placebo-controlled, parallel-group study. Clin Ther 2008;30(8):1448–60. 10.1016/j.clinthera.2008.08.006 18803987

[5] Drucker DJ, Nauck MA. The incretin system: glucagon-like peptide-1 receptor agonists and dipeptidyl peptidase-4 inhibitors in type 2 diabetes. Lancet 2006;368(9548):1696–705. 17098089 10.1016/S0140-6736(06)69705-5

[6] DeFronzo RA, Okerson T, Viswanathan P, Guan X, Holcombe JH, MacConell L. Effects of exenatide versus sitagliptin on postprandial glucose, insulin and glucagon secretion, gastric emptying, and caloric intake: a randomized, cross-over study. Curr Med Res Opin 2008;24(10):2943–52. 10.1185/03007990802418851 18786299

[7] Kelly AS, Metzig AM, Rudser KD, Fitch AK, Fox CK, Nathan BM, et al Exenatide as a weight-loss therapy in extreme pediatric obesity: a randomized, controlled pilot study. Obesity (Silver Spring) 2012;20(2):364–70. 22076596 10.1038/oby.2011.337 PMC3684414

[8] Dushay J, Gao C, Gopalakrishnan GS, Crawley M, Mitten EK, Wilker E, et al Short-term exenatide treatment leads to significant weight loss in a subset of obese women without diabetes. Diabetes Care 2012;35(1):4–11. 10.2337/dc11-0931 22040840 PMC3241299

[9] Astrup A, Carraro R, Finer N, Harper A, Kunesova M, Lean ME, et al Safety, tolerability and sustained weight loss over 2 years with the once-daily human GLP-1 analog, liraglutide. Int J Obes (Lond) 2012;36(6):843–54. 21844879 10.1038/ijo.2011.158 PMC3374073

[10] Kendall DM, Riddle MC, Rosenstock J, Zhuang D, Kim DD, Fineman MS, et al Effects of exenatide (exendin-4) on glycemic control over 30 weeks in patients with type 2 diabetes treated with metformin and a sulfonylurea. Diabetes Care 2005;28(5):1083–91. 15855571 10.2337/diacare.28.5.1083

[11] Hoenig M, Pach N, Thomaseth K, Le A, Schaeffer D, Ferguson DC. Cats differ from other species in their cytokine and antioxidant enzyme response when developing obesity. Obesity (Silver Spring) 2013;21(9):E407–14. 23408676 10.1002/oby.20306

[12] Appleton DJ, Rand JS, Sunvold GD. Insulin sensitivity decreases with obesity, and lean cats with low insulin sensitivity are at greatest risk of glucose intolerance with weight gain. J Feline Med Surg 2001;3(4):211–28. 11795959 10.1053/jfms.2001.0138 PMC10822293

[13] Courcier EA, O'Higgins R, Mellor DJ, Yam PS. Prevalence and risk factors for feline obesity in a first opinion practice in Glasgow, Scotland. J Feline Med Surg 2010;12(10):746–53. 10.1016/j.jfms.2010.05.011 20685143 PMC11135528

[14] Kley S, Hoenig M, Glushka J, Jin ES, Burgess SC, Waldron M, et al The impact of obesity, sex, and diet on hepatic glucose production in cats. Am J Physiol Regul Integr Comp Physiol 2009;296(4):R936–43. 10.1152/ajpregu.90771.2008 19193946 PMC2698604

[15] Appleton DJ, Rand JS, Sunvold GD. Plasma leptin concentrations are independently associated with insulin sensitivity in lean and overweight cats. J Feline Med Surg 2002;4(2):83–93. 12027507 10.1053/jfms.2002.0166 PMC10822654

[16] Bjornvad CR, Rand JS, Tan HY, Jensen KS, Rose FJ, Armstrong PJ, et al Obesity and sex influence insulin resistance and total and multimer adiponectin levels in adult neutered domestic shorthair client-owned cats. Domest Anim Endocrinol 2014;47:55–64. 10.1016/j.domaniend.2013.11.006 24373250

[17] Gilor C, Graves TK, Gilor S, Ridge TK, Rick M. The GLP-1 mimetic exenatide potentiates insulin secretion in healthy cats. Domest Anim Endocrinol 2011;41(1):42–9. 10.1016/j.domaniend.2011.03.001 21645806

[18] Seyfert TM, Brunker JD, Maxwell LK, Payton ME, McFarlane D. Effects of a Glucagon-like Peptide-1 Mimetic (Exenatide) in Healthy Cats. Intern J Appl Res Vet Med 2012;10(2):147–56.

[19] Hall MJ, Adin CA, Borin-Crivellenti S, Rudinsky AJ, Rajala-Schultz P, Lakritz J, et al Pharmacokinetics and pharmacodynamics of the glucagon-like peptide-1 analog liraglutide in healthy cats. Domest Anim Endocrinol 2015;51:114–21. 10.1016/j.domaniend.2014.12.001 25625650

[20] Nelson RW, Reusch CE. Animal models of disease: classification and etiology of diabetes in dogs and cats. J Endocrinol 2014;222(3):T1–9. 10.1530/JOE-14-0202 24982466

[21] Goossens MM, Nelson RW, Feldman EC, Griffey SM. Response to insulin treatment and survival in 104 cats with diabetes mellitus (1985–1995). J Vet Intern Med 1998;12(1):1–6. 9503353 10.1111/j.1939-1676.1998.tb00489.x

[22] Michiels L, Reusch CE, Boari A, Petrie G, Mandigers P, Thollot IG, et al Treatment of 46 cats with porcine lente insulin—a prospective, multicentre study. J Feline Med Surg 2008;10(5):439–51. 10.1016/j.jfms.2007.10.013 18619886 PMC11271226

[23] Laflamme D. Development and Validation of a Body Condition Score System for Cats: A Clinical Tool. Feline Practice 1997;25(5–6):13–8.

[24] Byetta: EPAR—Product Information. Annex I: Summary of Product Characteristics. European Medicines Agency 2009. Available: http://www.ema.europa.eu/docs/en_GB/document_library/EPAR_-_Product_Information/human/000698/WC500051845.pdf

[25] Padrutt I, Lutz TA, Reusch CE, Zini E. Effects of the glucagon-like peptide-1 (GLP-1) analogues exenatide, exenatide extended-release, and of the dipeptidylpeptidase-4 (DPP-4) inhibitor sitagliptin on glucose metabolism in healthy cats. Res Vet Sci 2015;99:23–9. 10.1016/j.rvsc.2014.12.001 25648286

[26] Slingerland LI, Robben JH, van Haeften TW, Kooistra HS, Rijnberk A. Insulin sensitivity and beta-cell function in healthy cats: assessment with the use of the hyperglycemic glucose clamp. Horm Metab Res 2007;39(5):341–6. 17533575 10.1055/s-2007-976541

[27] Link KR, Rand JS. Reference values for glucose tolerance and glucose tolerance status in cats. J Am Vet Med Assoc 1998;213(4):492–6. 9713530

[28] Wewer Albrechtsen NJ, Hartmann B, Veedfald S, Windelov JA, Plamboeck A, Bojsen-Moller KN, et al Hyperglucagonaemia analysed by glucagon sandwich ELISA: nonspecific interference or truly elevated levels? Diabetologia 2014;57(9):1919–26. 10.1007/s00125-014-3283-z 24891019

[29] Wewer Albrechtsen NJ, Bak MJ, Hartmann B, Christensen LW, Kuhre RE, Deacon CF, et al Stability of glucagon-like peptide 1 and glucagon in human plasma. Endocri Connect 2015;4(1):50–7. 10.1530/EC-14-0126 PMC4317691 25596009

[30] Simonsen L, Holst JJ, Madsen K, Deacon CF. The C-terminal extension of exendin-4 provides additional metabolic stability when added to GLP-1, while there is minimal effect of truncating exendin-4 in anaesthetized pigs. Regul Pept 2013;181:17–21. 10.1016/j.regpep.2012.12.012 23318502

[31] Backus RC, Havel PJ, Gingerich RL, Rogers QR. Relationship between serum leptin immunoreactivity and body fat mass as estimated by use of a novel gas-phase Fourier transform infrared spectroscopy deuterium dilution method in cats. Am J Vet Res 2000;61(7):796–801. 10895903 10.2460/ajvr.2000.61.796

[32] Tvarijonaviciute A, German AJ, Martinez-Subiela S, Tecles F, Ceron JJ. Analytical performance of commercially-available assays for feline insulin-like growth factor 1 (IGF-1), adiponectin and ghrelin measurements. J Feline Med Surg 2012;14(2):138–46. 10.1177/1098612X11432236 22314090 PMC10822485

[33] Rudinsky AJ, Adin CA, Borin-Crivellenti S, Rajala-Schultz P, Hall MJ, Gilor C. Pharmacology of the glucagon-like peptide-1 analog exenatide extended-release in healthy cats. Domest Anim Endocrinol 2015;51:78–85. 10.1016/j.domaniend.2014.12.003 25594949

[34] Nelson RW, Himsel CA, Feldman EC, Bottoms GD. Glucose tolerance and insulin response in normal-weight and obese cats. Am J Vet Res 1990;51(9):1357–62. 2204297

[35] Knop FK, Aaboe K, Vilsboll T, Volund A, Holst JJ, Krarup T, et al Impaired incretin effect and fasting hyperglucagonaemia characterizing type 2 diabetic subjects are early signs of dysmetabolism in obesity. Diabetes Obes Metab 2012;14(6):500–10. 10.1111/j.1463-1326.2011.01549.x 22171657

[36] Fineman MS, Mace KF, Diamant M, Darsow T, Cirincione BB, Booker Porter TK, et al Clinical relevance of anti-exenatide antibodies: safety, efficacy and cross-reactivity with long-term treatment. Diabetes Obes Metab 2012;14(6):546–54. 10.1111/j.1463-1326.2012.01561.x 22236356

[37] Xu G, Stoffers DA, Habener JF, Bonner-Weir S. Exendin-4 stimulates both beta-cell replication and neogenesis, resulting in increased beta-cell mass and improved glucose tolerance in diabetic rats. Diabetes 1999;48(12):2270–6. 10580413 10.2337/diabetes.48.12.2270

[38] Brooks D, Churchill J, Fein K, Linder D, Michel KE, Tudor K, et al 2014 AAHA weight management guidelines for dogs and cats. J Am Anim Hosp Assoc 2014;50(1):1–11. 24216501 10.5326/JAAHA-MS-6331

[39] Appleton DJ, Rand JS, Sunvold GD. Plasma leptin concentrations in cats: reference range, effect of weight gain and relationship with adiposity as measured by dual energy X-ray absorptiometry. J Feline Med Surg 2000;2(4):191–9. 11716618 10.1053/jfms.2000.0103 PMC10829137

[40] O'Brien TD, Hayden DW, Johnson KH, Stevens JB. High dose intravenous glucose tolerance test and serum insulin and glucagon levels in diabetic and non-diabetic cats: relationships to insular amyloidosis. Vet Pathol 1985;22(3):250–61. 3890345 10.1177/030098588502200308

[41] Kolterman OG, Buse JB, Fineman MS, Gaines E, Heintz S, Bicsak TA, et al Synthetic exendin-4 (exenatide) significantly reduces postprandial and fasting plasma glucose in subjects with type 2 diabetes. J Clin Endocrinol Metab 2003;88(7):3082–9. 12843147 10.1210/jc.2002-021545

[42] Drucker DJ, Buse JB, Taylor K, Kendall DM, Trautmann M, Zhuang D, et al Exenatide once weekly versus twice daily for the treatment of type 2 diabetes: a randomised, open-label, non-inferiority study. Lancet 2008;372(9645):1240–50. 10.1016/S0140-6736(08)61206-4 18782641

[43] Lund A, Bagger JI, Christensen M, Knop FK, Vilsboll T. Glucagon and type 2 diabetes: the return of the alpha cell. Curr Diab Rep 2014;14(12):555 10.1007/s11892-014-0555-4 25344790

[44] Safety Alert on Human Medical Products. Byetta (exenatide) October 2007. U.S. Food and Drug Administration. 2007. Available: http://www.fda.gov/Safety/MedWatch/SafetyInformation/SafetyAlertsforHumanMedicalProducts/ucm150839.htm.

[45] Li L, Shen J, Bala MM, Busse JW, Ebrahim S, Vandvik PO, et al Incretin treatment and risk of pancreatitis in patients with type 2 diabetes mellitus: systematic review and meta-analysis of randomised and non-randomised studies. BMJ 2014;348:g2366 10.1136/bmj.g2366 24736555 PMC3987051

